# Serum Vitamin D Concentration ≥75 nmol/L Is Related to Decreased Cardiometabolic and Inflammatory Biomarkers, Metabolic Syndrome, and Diabetes; and Increased Cardiorespiratory Fitness in US Adults

**DOI:** 10.3390/nu12030730

**Published:** 2020-03-10

**Authors:** Vijay Ganji, Vin Tangpricha, Xu Zhang

**Affiliations:** 1 Human Nutrition Department, College of Health Sciences, QU Health, Qatar University, Doha P.O. Box 2713, Qatar; 2 Division of Endocrinology, Metabolism and Lipids, Department of Medicine, Emory University, Atlanta, GA 30322, USA; vin.tangpricha@emory.edu; 3 Atlanta VA Medical Center, Decatur, GA 30300, USA; 4 University of Texas Health Center Houston, Houston, TX 77030, USA; Xu.Zhang@uth.tmc.edu

**Keywords:** cardiometabolic diseases, cardiorespiratory fitness, diabetes, metabolic syndrome, National Health and Nutrition Examination Surveys, USA, vitamin D

## Abstract

A serum vitamin D [25-hydroxyvitamin D, 25(OH)D] concentration of ≥75 nmol/L is recommended for optimal health. We investigated the relationship between serum 25(OH)D and metabolic syndrome (MetS), diabetes, cardiometabolic biomarkers, and cardiorespiratory fitness (CRF) in US adults using clinical cut points recommended by health organizations. Data from USA’s National Health and Nutrition Examination Surveys were used. Prevalences and likelihood of having MetS and diabetes according to clinical cut points for serum 25(OH)D (<30 nmol/L, 30-<50 nmol/L, 50-<75 nmo/L, and ≥75 nmol/L) were determined with multivariate logistic regression. Relations between serum 25(OH)D and various cardiometabolic biomarkers, CRF, MetS, and diabetes were tested using multivariable adjusted regression. Prevalence of MetS and diabetes were significantly lower in individuals with serum 25(OH)D ≥75 nmol/L (MetS, 21.6%; diabetes, 4.1%) compared to those with 25(OH)D <30 nmol/L (MetS, 45.5%; diabetes, 11.6%) (*p* < 0.0001). Individuals with serum 25(OH)D ≥75 nmol/L had significantly lower waist circumference (*p* < 0.0001), C-reactive protein (*p* = 0.003), glycated hemoglobin (*p* < 0.0002), fasting triglycerides (*p* < 0.0001), total homocysteine (*p* < 0.0001), and insulin resistance (*p* = 0.0001) and had significantly higher HDL-cholesterol (*p* < 0.0001) and maximal oxygen uptake (marker for CRF) (*p*< 0.0009) compared to those with 25(OH)D <30 nmol/L. In conclusion, serum 25(OH)D ≥75 nmol/L is associated with positive indicators related to cardiometabolic diseases in US adults.

## 1. Introduction

Serum vitamin D [25-hydroxyvitamin D, 25(OH)D] concentration is used as a biomarker of vitamin D status. 25(OH)D in the circulation represents endogenously synthesized vitamin D in the dermis and from diet and supplements. Recently there has been an increased interest in vitamin D due to widespread prevalence of suboptimal serum 25(OH)D concentrations in the US and across the globe. The prevalence of hypovitaminosis D [<50 nmol/L of 25(OH)D] was ≈32% in the US population [1]. Based on the recent data from the National Health and Nutrition Examination Survey (NHANES), there has been an improvement in serum 25(OH)D concentrations in the US population since 2006 [2]. Potential explanations for vitamin D insufficiency are decreased outdoor physical activity, limited dietary sources, increased obesity and overweight, and increased use of sunblock lotions [3,4,5].

The role of vitamin D in bone metabolism is well documented [6]. Recent evidence links vitamin D to a wide range of infirmities such as metabolic syndrome (MetS), cardiovascular disease (CVD) [7], diabetes mellitus [8], hypertension [9], depression [10], and certain types of cancer [11]. Known cardiometabolic risk factors are MetS, diabetes, insulin resistance, elevated inflammatory markers, and declined cardiorespiratory fitness (CRF). MetS is characterized by increased abdominal adiposity, perturbations in circulating lipids, deranged glucose metabolism, increased blood pressure, insulin resistance, and elevated inflammatory markers [12]. In the US, the prevalence of MetS has risen from 6.4% in 1999–2000 to 8.6% in 2001–2006 [13]. MetS is a precursor for type-2 diabetes [14].

Recent evidence suggests that serum 25(OH)D is related to skeletal muscle function and physical performance [15]. It has been reported that decreased maximal oxygen uptake (VO_2_ max), a marker of cardio-respiratory fitness (CRF) is associated with increased risk for CVD [16]. Studies on the relationship between vitamin D and CRF yielded conflicting results [17,18,19]. Ardestani et al. [17] and Mowry et al. [18] reported that higher serum 25(OH)D concentration was related to higher peak VO_2_ max, while Carson et al. [19] reported no relation between serum 25(OH)D and CRF in boys. It is not clearly known whether serum vitamin D deficiency is related to low CRF in the US adult population.

The biological function of vitamin D in inflammation is unclear. Studies linking vitamin D with inflammation have also yielded equivocal findings [20,21]. A few studies reported a relation between serum 25(OH)D concentration and metabolic diseases [22,23]. Previous studies relating serum 25(OH)D with cardiometabolic disease were based on artificial quintile/quartile/tertile vitamin D cut off values [7,22]. We tested the hypothesis is that a serum 25(OHD) concentration of ≥75 nmol/L is associated with decreased cardiometabolic and inflammatory markers, decreased prevalence of MetS and diabetes, and increased CRF. In this study we used clinical cut off values for serum 25(OH)D recommended by the Endocrine Society (ES) [24] and the Institute of Medicine (IOM) [25]. Therefore, the objective of this study was to investigate the relationship between serum 25(OH)D and several cardiometabolic biomarkers, MetS, diabetes, and CRF based on the clinical cut off values for serum 25(OH)D using the data from National Health and Nutrition Examination Surveys (NHANES), 2001–2002, 2003–2004, and 2005–2006.

## 2. Subjects and Methods

### 2.1. Brief Survey Description and Setting

The study design is cross-sectional in nature on a large civilian US adult population. NHANES are conducted by the National Center for Health Statistics (NCHS) on the US population. NHANES represent the general US population because these surveys were based on a stratified, multistage, probability sample design. Beginning 1999, NHANESs were conducted on a yearly basis and the data are posted on the NHANES website in 2-year cycles for public at large. Subjects were asked to provide data on their diet, demographic characteristics, and health status. This information was collected from face-to-face interviews. Subjects gave blood and urine samples in the Mobile Examination Center (MEC). Also, physical examination was conducted in MEC. To achieve reliable data, certain populations such as low-income individuals, adolescents, subjects ≥60 years old, and minorities such as non-Hispanic black (NHB) and Hispanics/Mexican American (H/MA) were over sampled to achieve reliable estimates for these populations. For this study, we used 3 cycles of NHANESs (2001 to 2006). NHANES 2001–2002, 2003–2004, and 2005–2006 were conducted from January 2001 to December 2002 (*n* = 11039; 10,477 were examined in MECs), January 2003 to December 2004 (*n* = 12761; 9643 were examined in MECs), and January 2005 to December 2006 (*n* = 12862; 9950 were examined in MECs), respectively. Data were collected from November 1 through April 30 in the South and from May 1 through October 31 in the North to protect MECs from damage from inclement weather. NCHS’s Human Subjects Ethics Review Committee approved all NHANESs study protocols. All subjects consented before participation in the survey. NCHS conducted study accordance with the Declaration of Helsinki. The detailed information on ethics of the NHANES is publically available at https://www.cdc.gov/nchs/nhanes/nhanes2009–2010/current_nhanes_09_10.htm (NCHS Ethics Review Board Protocol Number for the NHANES 2005–2010 is #2005–2006 and for the NHANES 1999–2004 is #98–12). The description of the survey methods was reported in detail elsewhere [26].

### 2.2. Sample Derivation and Eligibility Criteria

**Figure 1:**
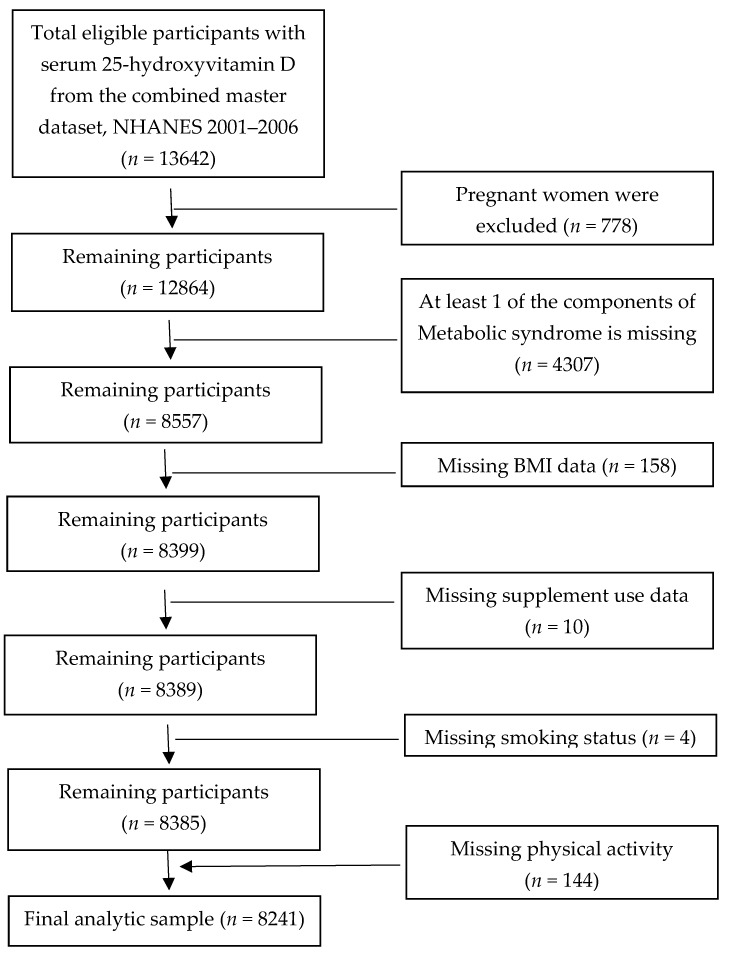
Strengthening of Reporting of Observational Studies in Epidemiology (STROBE) flow chart-sample derivation for the association between serum 25-hydroxyvitamin D concentration and prevalence of metabolic syndrome.

Participants >19 years old from NHANES 2001–2002, 2003–2004, and 2005–2006 were included in this study. These 3 data sets were combined into one master data set, NHANES 2001–2006. A total of 13,642 participants had measured circulating 25(OH)D concentrations. Of those, ≈51% men and ≈49% women. The mean age of the study population was ≈51 years old. The mean BMI was ≈28 kg/m^2^. After excluding pregnant women (*n* = 778) and subjects with missing data for at least one component of MetS status (*n* = 4307), BMI (*n* = 158), consumption of supplements (*n* = 10), smoking status (*n* = 4), and physical activity (*n* = 144), the final analytic sample was 8241, yielding the weighted population size of 1,214,884,618. This sample formed the basis for the analysis of the association between 25(OH)D and MetS prevalence (Figure 1). 

**Figure 2:**
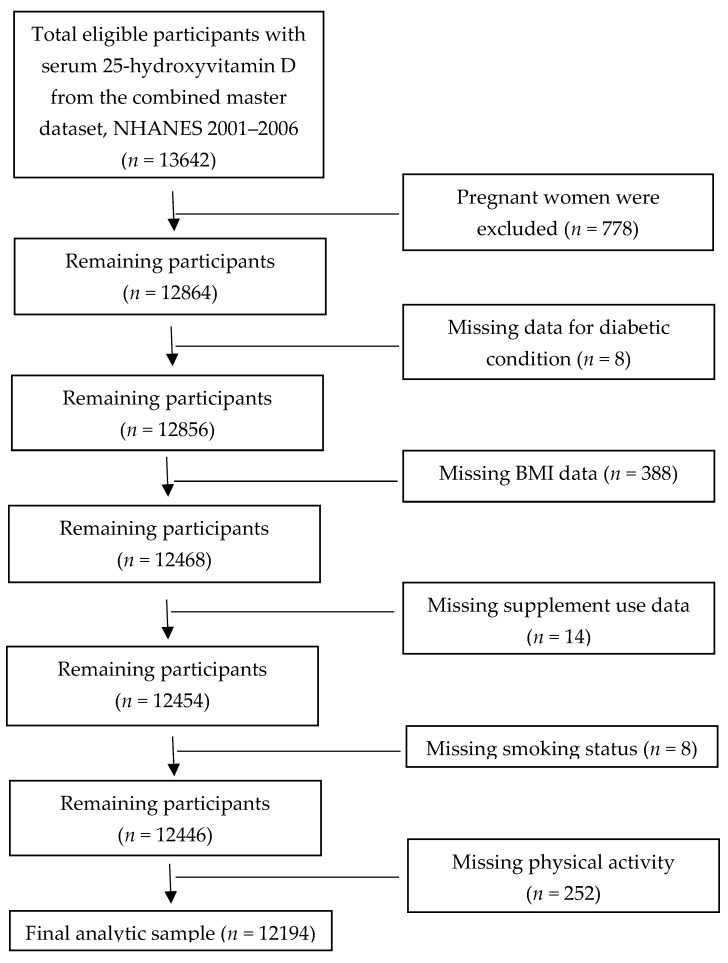
Strengthening of Reporting of Observational Studies in Epidemiology (STROBE) flow chart-sample derivation for the association between serum 25-hydroxyvitamin D concentration and diabetes risk.

Sample size for the assessment of association between serum 25(OH)D concentration and diabetes risk was 12,194 (weighted population size of 182,793,002). The initial sample for this analysis was 13,642 participants with reported serum 25(OH)D concentrations for the combined 3 cycles of NHANESs (2001–2006). Of those, 1448 subjects were sequentially excluded to derive the analytic sample. These were pregnant women (*n* = 778) and missing participant data for diabetic condition (*n* = 8), BMI (*n* = 388), consumption of supplements (*n* = 14), smoking status (*n* = 8), and physical activity (*n* = 252) (Figure 2).

Sample sizes for the assessment of relationship between serum 25(OH)D and several individual cardiometabolic risk factors varied. Out of 14,542 adults examined in MEC, we applied the similar set of exclusion criteria sequentially (as described above). Exclusion occurred if serum 25(OH)D concentrations were missing, a woman was pregnant, and/or cardiometabolic responses were missing. A subject was also excluded if the status on any of the major risk factors for a particular cardiometabolic biomarker was unknown (BMI, use of supplement, smoking, physical activity). The sample sizes for cardiometabolic risk factors varied (Table 1). Generally, the cardiometabolic biomarker responses requiring fasting blood measurements had smaller sample sizes because MEC-examined subjects were selected to give fasting blood test. The measurements for fibrinogen and VO_2_ max were available only for 1253 and 2625 subjects, respectively, because these biomarkers were measured in only one 2-year NHANES cycle. 

### 2.3. Criteria for MetS Diagnosis 

In this study, the MetS in adults was defined according to the National Cholesterol Education Program-Adult Treatment Panel III [27]. Accordingly, the MetS was defined as having at least 3 of the following cardimetabolic risk factors: (1) waist circumference >102 cm for men and >88 cm for women, (2) triglycerides ≥150 mg/dL, (3) serum HDL cholesterol <40 mg/dL for men and <50 mg/dL for women, (4) either systolic blood pressure >130 mm Hg or diastolic blood pressure >85 mm Hg, and (5) fasting glucose ≥100 mg/dL. Individuals who had diagnosis for high blood pressure or using medications for high blood pressure were considered meeting the criteria for elevated blood pressure. Individuals with diagnosed diabetes or using insulin or oral hypoglycemic medications were considered meeting the criteria for high blood glucose. A case of MetS phenotype was determined if at least 3 out of 5 above mentioned criteria were present.

### 2.4. Description of Study Variables

Several potential confounding variables such as race-ethnicity, age, gender, BMI, socioeconomic status (poverty income ratio (PIR)), consumption of supplements, alcohol consumption, smoking, physical activity, and season of blood draw were included in the data analysis. Age and BMI were used as continuous variables. Participants’ self-reported race-ethnicity as non-Hispanic white (NHW), NHB, H/MA, and others (individuals of mixed or multiple race). PIR is the total family income divided by the family’s poverty threshold. The PIR was classified into three groups, i.e., <1.0 (below poverty), 1.0–2.5 (middle income), and ≥2.5 (higher income). Individuals who did not have data for PIR were placed into “not reported” category. 

NHANES’s examination component was the source for waist circumference, BMI, and blood pressure. Measurement of waist circumference has been described in the NHANES protocols [28]. Blood pressure was measured with a mercury sphygmomanometer [29]. Subjects who answered ‘yes’ to the question “did you take supplements in the past 30 day” were considered as users of supplements. Smoking status was defined as “yes” if the adult was smoking cigarettes. Alcohol intake was considered at 4 levels, i.e., amount unreported, small amount (<2 drinks), 2–3 drinks, and >3 drinks on the days that one drank. Physical activity was determined to be active if a participant engaged in vigorous/moderate activities or muscle strengthening activities in the past 30 days.

### 2.5. Description of Biochemical Assessments

Blood was collected by venipuncture according to the standard procedures. Serum 25(OH)D concentration was measured at the National Center for Environmental Health, CDC, Atlanta, GA (Diasorin RIA kit assay, Stillwater, MN, USA). The heparin manganese precipitation method was used in NHANES 2001–2002 and an immunoassay method was used in NHANES 2005–2006 for serum HDL-cholesterol measurement. An enzymatic method was used to measure the serum triglycerides (Johns Hopkins Hospital, Baltimore, MD, USA). In NHANES 2001–2002, an enzymatic method and in NHANES 2003–2006 the hexokinase method was used to measure the glucose. Because of assay changes between NHANES 2005–2006 and 2003–2004, the following regression method (Equation (1)) was used to normalize the glucose concentrations as per NCHS guidelines.

Fasting blood glucose _NHANES 2005–2006_ = (0.9815 × Fasting blood glucose _NHANES 2003–2004_) + 3.5707 (1)

In NHANES 2001–2002, 2003–2004, and 2005–2006, serum insulin concentration was measured with the Pharmacia method, Tosh method (2-site immuneenzymometric assay), and Mercodia ELISA immunoassay method, respectively. Because the methods for insulin has changed among the three cycles of NHANES and for the insulin concentration to be valid when NHANESs data were merged, as per NCHS guidelines, the following linear regression method (Equation (2)) was used to adjust the NHANES 2001–2002 serum insulin to make it comparable to 2003–2004 values:

Serum insulin _NHANES 2003–2004_ = (1.0027 × Serum insulin _NHANES 2001–2002_) − 2.2934 (2)

As per the NCHS guidelines, the following regression equation (Equation (3)) was used in order for the combined (NHANES 2005–2006 and NHANES 2003–2004) insulin concentrations to be valid in the analysis.

Serum insulin _NHANES 2003–2004_ = (1.0526 × Serum insulin _NHANES 2005–2006_) − 1.5674 (3)

Fasting glucose and serum insulin were measured in NHANES 2001–2004 at the Diabetes Diagnostic Laboratory, University of Missouri, USA and in NHANES 2005–2006 at the Fairview Medical Center Laboratory, University of Minnesota, Minneapolis. Insulin resistance was assessed with homeostatic model assessment-insulin resistance (HOMA-IR) (fasting insulin (µU/mL) × fasting glucose (mg/dL)/405) [30]. The latex-enhanced nephelometry method was used for serum C-reactive protein at University of Washington, Seattle. 

### 2.6. Statistical Analysis

Six-year sample weights were used to generate statistically reliable data estimates taking the NHANES complex survey method (multistage, probability design) into account, as per NCHS guidelines. The Taylor Linearization method was used to estimate variance. Detailed descriptions on sample weights and variance estimation methods were defined in the NHANES Analytic Guidelines [26]. Data analyses were performed with Statistical Analysis System-compatible SUDAAN (version 10.0.1; Research Triangle Institute, RT Park, NC, USA) in conjunction with Statistical Analysis System (version 9.2, Statistical Analysis System Institute Inc., Cary, NC, USA) statistical packages.

Significant differences between men and women for various cardiometabolic biomarkers and CRF were tested with a 2-tailed *t*-test. Because serum 25(OH)D, triglycerides, glucose, glycohemoglobin, insulin, C-peptide, C-reactive protein, HOMA-IR, and homocysteine concentrations were skewed, logarithm transformed values were used to satisfy the normality. Serum 25(OH)D concentrations were stratified into <30 nmol/L, 30-<50 nmol/L, 50-<75 nmo/L, and ≥75 nmol/L clinical cut off categories. Proportion of subjects with MetS and diabetes across the 4 clinical cut off points of serum 25(OH)D concentrations were compared with the Rao-Scott χ^2^ test. Relation between MetS prevalence and diabetes and serum 25(OH)D was determined with unadjusted and multivariable cumulative logistic regression analysis.

Multivariable adjusted odds ratios (OR) and 95% confidence intervals (CI) were determined for the occurrence of MetS and diabetes for each serum 25(OH)D clinical cut point after adjusting the data analysis for race-ethnicity, age, gender, BMI, and PIR. Interaction terms for the above mentioned variables were also included in the model. ORs for serum 25(OH)D <30 nmol/L, 30-<50 nmol/L, and 50-<75 nmol/L in relation to serum 25(OH)D ≥75 nmol/L (referent category) were compared. Significance between reference category and other categories was determined after adjusting the *p*-values for the multiple comparisons using Bonferroni correction. A *p*-value of <0.0083 (0.05/6) was considered significant between referent category and other categories in multiple comparison analysis. We have presented the results without gender stratification because gender was not significant in regression models that investigated the relation between serum 25(OH)D concentrations and MetS and diabetes.

The adjusted means for waist circumference, fasting glucose, glycated hemoglobin, triglycerides, blood pressure, HDL-cholesterol, LDL-cholesterol, serum insulin, C-peptide, HOMA-IR, C-reactive protein, total homocysteine, fibrinogen, and VO_2_ max across serum 25(OH)D concentration cut off values (as described before) were generated using multivariable regression. Models were adjusted for race-ethnicity, gender, age, consumption of supplements, season of blood draw, BMI, PIR, physical activity, smoking status, and alcohol consumption. Individual multivariable regression models were generated to establish the relation between 25(OH)D concentrations cut off values and waist circumference, fasting blood glucose, glycated hemoglobin, HDL-cholesterol, LDL-cholesterol, triglyceride, systolic blood pressure, diastolic blood pressure, C-reactive protein, serum insulin, C-peptide, HOMA-IR, total homocysteine, fibrinogen, and VO_2_ max. Natural logarithmic transformation was used to satisfy the normality requirement for fasting glucose, triglycerides, C-reactive protein, and HOMA-IR variables because these variables were found to be not normal. Multiple mean comparisons were performed to determine the significant differences between the multivariable adjusted means for serum 25(OH)D cut point categories for individual cardiometabolic biomarker with Bonferroni correction (0.05/6 comparisons = *p* < 0.0083). Statistical interactions between 25(OH)D concentrations and confounding variables were determined and these interaction terms were included in the data analysis. In all analyses a *p*-value of ≤0.05 was considered statistically significant.

## 3. Results

### 3.1. Baseline Characteristics

The study population characteristics are described in Table 1. Serum 25(OH)D concentrations were significantly higher in men than in women (*p* < 0.0003). Cardiometabolic biomarkers such as waist circumference, VO_2_ max, systolic and diastolic blood pressures, fasting triglycerides, LDL-cholesterol, fasting plasma glucose, serum insulin, serum C-peptide, HOMA-IR, and total homocysteine concentrations were significantly higher and HDL-cholesterol, C-reactive protein, and fibrinogen concentrations were significantly lower in men compared to women (*p* < 0.05).

The proportion of NHW was more and the proportion of NHB was less in the highest cut point compared to the lowest cut point of serum 25(OH)D concentrations. There were more subjects with obesity (based on the BMI) in the lowest clinical cut point compared to the highest serum 25(OH)D cut point. In the highest serum 25(OH)D cut point category, more subjects consumed supplements compared to the lowest serum 25(OH)D cut point category. Subjects surveyed in the summer were more in the highest cut point group compared to the lowest serum 25(OH)D cut point group (*p*< 0.05).

### 3.2. Serum 25(OH)D Concentrations, MetS and Diabetes

The association between serum 25(OH)D concentrations and prevalence of MetS is presented in Table 2. In US adults, the prevalence of MetS increased as the serum 25(OH)D concentrations decreased. The prevalence of MetS was significantly higher in individuals with serum 25(OH)D concentrations <30 nmol/L (45.5%) compared to individuals with serum 25(OH)D ≥75 nmol/L (21.6%) (*p* for trend <0.0001). In the multivariable adjusted logistic regression analysis, the odds of having MetS in subjects with serum 25(OH)D concentration <30 nmol/L is approximately 3 times higher (OR: 2.98; 95% CI: 2.98, 2.65; *p* for trend < 0.0001) compared to those with ES recommendation of serum 25(OH)D ≥75 nmol/L. 

The association between serum 25(OH)D concentrations and prevalence of diabetes is presented in Table 3. In US adults, the prevalence of diabetes increased as the serum 25(OH)D concentrations decreased. The prevalence of diabetes was significantly higher in individuals with serum 25(OH)D concentrations <30 nmol/L (11.6%) compared to individuals with serum 25(OH)D ≥75 nmol/L (4.1%) (*p* < 0.0001). In the multivariable adjusted logistic regression, the odds of having diabetes in individuals with serum 25(OH)D concentration <30 nmol/L was significantly higher (OR: 1.7; 95% CI: 1.19, 2.43; *p*-trend <0.008) compared to those with serum 25(OH)D concentration ≥75 nmol/L

### 3.3. Serum 25(OH)D Concentrations, Cardiometabolic Markers, and CRF

The association between serum 25(OH)D concentrations and various cardiometabolic risk factors is presented in Table 4. In the multivariable adjusted regression analysis, several cardiometabolic risk factors such as waist circumference, diastolic blood pressure, triglycerides, glycated hemoglobin, serum insulin, serum C-peptide, total homocysteine, C-reactive protein, and HOMA-IR were significantly higher in individuals with serum 25(OH)D concentrations <30 nmol/L compared to those with serum 25(OH)D concentrations ≥75 nmol/L. Whereas, VO_2_ max was significantly lower in individuals with serum 25(OH)D concentrations <30 nmol/L compared to those with serum 25(OH)D concentrations ≥75 nmol/L (*p* for trend 0.0009). No association was observed between serum 25(OH)D concentrations and LDL-cholesterol, fasting glucose, and fibrinogen.

## 4. Discussion

This is the first comprehensive study that specifically investigated the relation between serum 25(OH)D concentrations and a myriad of cardiometabolic risk factors using clinical cut off points as defined by the ES [24] and the IOM [25]. The ES defined serum 25(OH)D concentrations <50 nmol/L and 50 to <75 nmol/L as indicative of vitamin D deficiency and insufficiency, respectively. Also, the ES recommended ≥75 nmol/L serum 25(OH)D to maximize the benefit of vitamin D on bone health and non-calcemic functions [24]. The IOM defined <30 nmol/L, 30–50 nmol/L, and >50 nmol/L of serum 25(OH)D as indicative of deficiency, insufficiency, and sufficiency, respectively [25]. In this study using the data from large US national surveys, we report that individuals with serum 25(OH)D concentrations ≥75 nmol/L compared to those with <30 nmol/L had improved cardiometabolic biomarkers, decreased prevalences of MetS and diabetes, and increased VO_2_ max in the US adult population.

We found that serum 25(OH)D concentrations were significantly related to all individual markers of MetS and diabetes. The likelihood of having MetS and diabetes is linearly, inversely related to the serum 25(OH)D concentrations. The farther the below ≥75 nmol/L serum 25(OH)D, the greater the risk of having cardiometabolic diseases. For example, the risk for MetS is 78% higher in the 50-<75 nmol/L serum 25(OH)D group, 184% higher in the 30-<50 nmol/L serum 25(OH)D group, and 198% higher in the <30 nmol/L serum 25(OH)D group compared to the ≥75 nmol/L serum 25(OH)D group. The risk of having MetS in <75 nmol/L serum 25(OH)D concentrations groups (<30 nmol/L, 30-<50 nmol/L, and 50-<75 nmol/L) is by and large similar (overlapping 95% CI) (Table 2) suggesting that serum 25(OH)D should be at or above 75 nmol/L to be protective against MetS. Along these lines, the risk of having diabetes in the lower clinical cutoff serum 25(OH)D concentrations (<75 nmol/L) is similar (overlapping 95% CI) (Table 3). This also suggests that serum 25(OH)D needed to be at or above 75 nmol/L in order to have a beneficial impact on the risk for diabetes.

Earlier studies relating serum vitamin D with MetS yielded mixed results. Our results of low vitamin D relation with high prevalence of MetS is in agreement with recent findings [31,32]. Two studies on middle-aged population reported an inverse relationship between 25(OH)D and triglycerides and blood pressure [33,34]. Others observed a direct relationship with HDL-cholesterol [35]. In contrast, Huang et al. [36] found no relationship between serum 25(OH)D and MetS after adjusting for BMI and HOMA-IR. Although evidence exists linking low vitamin D with higher cardiometabolic biomarkers, randomized controlled trials (RCTs) are warranted to determine the intake level of vitamin D that reduces the risk of cardiometabolic diseases. 

We found individuals with <30 nmol/L of serum 25(OH)D have ≈183% higher diabetes prevalence compared to those with ≥75 nmol/L 25(OH)D. This translates into 70% increased risk of having diabetes in those with <30 nmol/L serum 25(OH)D compared to those with ≥75 nmol/L serum 25(OH)D. In a recent meta-analysis based on 28,258 older subjects, Lucato et al. [37] found that hypovitaminosis D was associated with incident diabetes (17%) at follow up after adjusting for confounding variables. The mechanism through which vitamin D modulates glucose homeostasis and the risk of diabetes is not clear. However, emerging evidence points to a definitive pathway through which vitamin D affects glucose metabolism. Because vitamin D receptors (VDR) are present on β-cells of pancreas [38], a role for vitamin D in glucose homeostasis has been proposed. VDR deficient mice exhibited poor glucose tolerance and decreased insulin concentration [39]. Further, Sisley et al. [40] demonstrated a direct role of vitamin D and VDRs in glucose metabolism in rodents. Administration of 1,25-dihydroxyvitamin D [1,25(OH)_2_D], a hormone form, into the 3rd ventricle of the brain improved glucose tolerance and hepatic insulin sensitivity through VDR action [40]. They also demonstrated that administration of 1,25(OH)_2_D into the brain dramatically decreased the food intake and body weight in obese rodents. Although a clear cause and effect for vitamin D in glucose and insulin metabolism and body weight has been established, it is surprising that studies on vitamin D supplementation and CVD have yielded inconsistent results [41,42,43,44,45,46,47].

We report, for the first time in a large US sample that individuals with ≥75 nmol/L 25(OH)D had ≈5.9% higher VO_2_ max (a marker of CRF) compared to those with <30 nmol/L. Growing evidence links low CRF to increased risk for CVD and cancer [16]. Koundourakis et al. [48] reported a linear relationship between vitamin D and VO_2_ max in professional soccer players. Further Lammle et al. [49] found a direct association between serum vitamin D and aerobic performance in adolescents. It is possible that the association between vitamin D and VO_2_ max relates to the protective effect of vitamin D on lung function [48]. Adolescent boys who had higher vitamin D (>51 nmol/L) had higher muscle strength compared to those with <31 nmol/L [19]. Although our findings confirm the direct relationship between serum 25(OH)D and VO_2_ max, the difference in VO_2_ max we observed between the highest and the lowest serum 25(OH)D concentrations cut points was ≈1.9 mL/kg/min. Whether this small difference in VO_2_ max has any clinical relevance is not known. Controlled studies are needed to establish a cause and effect relation between vitamin D and CRF.

High circulating acute phase inflammatory markers, i.e., C-reactive protein and fibrinogen have been linked to chronic inflammatory diseases such as MetS, diabetes, obesity, and CVD [50,51,52]. Using the clinical cutoff values for serum 25(OH)D concentrations, we report that subjects with low serum 25(OH)D (<30 nmol/L) have high serum C-reactive protein. Individuals with serum ≥75 nmol/L 25(OH)D have ≈16.8% less C-reactive protein compared to those with serum <30 nmol/L 25(OH)D (*p* < 0.003). A recent meta-analysis demonstrated that vitamin D supplementation lowered C-reactive protein [53] suggesting that vitamin D is an anti-inflammatory agent [54]. The exact mechanism linking vitamin D with inflammation is not completely understood. Additionally, we found no relation between serum 25(OH)D and serum fibrinogen. This is may be due to small sample size because fibrinogen was measured in limited number of subjects in NHANES.

Although individuals with serum ≥75 nmol/L 25(OH)D had significantly lower risk of MetS, only the association between 25(OH)D and diastolic blood pressure was significant (*p* < 0.008). Individuals with ≥75 nmol/L 25(OH)D had ≈3.7% lower diastolic blood pressure than those with <30 nmol/L 25(OH)D. Support for an inverse association of 25(OH)D with blood pressure from epidemiological and prospective studies is very strong [55,56,57,58]. Because VDRs, 1,25(OH)_2_D, and 1-α hydroxylase are found in vascular endothelial cells, a function for vitamin D and VDRs in blood pressure has been proposed [59]. Deletion of the VDR gene in endothelial cells lead to endothelial dysfunction (impaired blood vessel relaxation) [60]. Additionally, vitamin D plays a role in renin-angiotensin-aldosterone system (RAAS), which regulates blood pressure. VDR null-mice exhibited increased concentrations of renin and angiotensin II leading to hypertension. In wild type mice, 1,25(OH)_2_D administration reduced the expression of renin [61,62]. Although the evidence from animal studies clearly links vitamin D to blood pressure, vitamin D supplementation studies on hypertension in humans yielded mixed results. Vitamin D_3_ (33000 IU) administration for every 2-weeks for six months reduced systolic blood pressure significantly [63]. A large meta-analysis of 81 studies by Mirhosseini et al. [64] reported that vitamin D improved blood pressure, dyslipidemia, and hypertension. A recent meta-analysis on 17 RCTs reported that vitamin D supplementation reduced systolic and diastolic pressures in vitamin D deficient and in hypertensive individuals [65]. In contrast, Golzarand et al. [66] reported no effect of vitamin D supplementation on blood pressure. Adequately powered, well-controlled, multicenter, prospective, RCTs are needed to disentangle the complex relationship between vitamin D and blood pressure.

The evidence from epidemiological studies [67], RCTs [68] and systematic reviews and meta-analysis of RCTs [42] linking vitamin D deficiency with insulin resistance is strong. However, in contrast, Poolsup et al. [42] reported no effect of vitamin D supplementation on HOMA-IR in a meta-analysis study that was based on ten RTCs. We confirm previous findings in this large nationally representative sample survey on US adults. In this study, we found that the HOMA-IR (a surrogate marker for insulin resistance) was ≈42% higher in the serum 25(OH)D <30 nmol/L group compared to the serum 25(OH)D ≥75 nmol/L group. In fact, the highest cut point group had significantly lower HOMA-IR compared to all lower cut point serum 25(OH)D groups, which suggests that persons with serum 25(OH)D ≥75 nmol/L are protected against insulin resistance-related pathologies. It is not very clear at what level of HOMA-IR a person is considered insulin resistant. Esteghamati et al. [69] reported a HOMA-IR cut off value of 1.775 for the diagnosis of MetS in Iranians. Another study from the same geographical region reported a cut point of 2.48 of HOMA-IR [70]. Arellano-Ruiz et al. [71] reported a HOMA-IR of 2.3 for the diagnosis of MetS. It is reasonable to assume that the HOMA-IR is between 1.775 and 2.48 for the diagnosis of MetS. Based on this HOMA-IR criteria, the risk of having MetS in the serum 25(OH)D ≥75 nmol/L group is very low to null (multivariable adjusted HOMA-IR is 1.76). On the other hand, subjects in the serum 25(OH)D <30 nmol/L group had the highest risk of having MetS. In this group, on average (multivariable adjusted HOMA-IR is 2.5), all subjects met the HOMA-IR criteria for the MetS. There is a need to establish a clinical cut point for HOMA-IR for the diagnosis of MetS in the US population.

Because this study was based on a nationally representative sample, these results are generalizable to population at large described in this study. Due to the cross-sectional nature of this study, results should not be viewed in terms of cause and effect relationships. Because of richness of NHANES data, we included several confounding variables for serum 25(OH)D concentration in the analysis. We were unable to separate the data by diabetes type 1 or type 2 status because NHANES survey did not contain that specific data. The MetS sample contains all diabetes cases who had met the MetS diagnostic criteria (please see 2.3 above). Therefore, there was an overlap of subjects between MetS and diabetes logistic regression analysis. Whether this overlap of diabetes cases in the MetS analysis lead to errors is not known. Overall, US adults with serum 25(OH)D <30 nmol/L have significantly higher prevalence of MetS and diabetes and higher inflammatory markers and reduced CRF compared to those in the serum 25(OH)D ≥75 nmol/L group. Recent evidence from controlled trials and meta-analysis is promising with regard to reporting beneficial effect of increased vitamin D in reducing the risk of cardiometabolic diseases. Our findings support the evidence that serum 25(OH)D concentrations ≥75 nmol/L are associated with improved metabolic pathologies related to inflammation, insulin resistance, MetS, and diabetes [72,73,74]. Given the role of vitamin D in various metabolic functions and increased prevalence of hypovitaminosis D in general population, more controlled studies are needed to determine whether serum 25(OH)D concentrations ≥75 nmol/L can be protective against cardiometabolic diseases.

## Figures and Tables

**Table 1:** Characteristics of study population: National Health and Nutrition Examination Surveys (NHANES), (2001–2006) ^1^.

Characteristic	Men	Women	*p*-Value ^2^
Serum 25(OH)D (nmol/L) (*n* = 8241) Body mass index (kg/m^2^) (*n* = 8241) Waist circumference (cm) (*n* = 8155)	55.5 ± 0.8 27.9 ± 0.1 99.4 ± 0.3	53.5 ± 0.9 27.8 ± 0.2 92.5 ± 0.4	0.0003 0.43 <0.0001
Systolic blood pressure (mm Hg) (*n* = 8020) Diastolic blood pressure (mm Hg) (*n* = 8020)	122 ± 0.4 72 ± 0.3	120 ± 0.4 69 ± 0.3	0.0003 <0.0001
Serum HDL-cholesterol (mg/dL) (*n* = 8231) Serum LDL-cholesterol (mg/dL) (*n* = 5243)	48 ± 0.3 114 ± 1	59 ± 0.4 111 ± 1	<0.0001 <0.02
Serum triglycerides (mg/dL) (*n* = 5404)	130 ± 2	110 ± 2	<0.0001
Plasma glucose (mg/dL) (*n* = 5415) Blood glycohemoglobin (%) (*n* = 8225)	101 ± 0.4 5.5 ± 0.01	97 ± 0.5 5.5 ± 0.02	<0.0001 0.33
Serum insulin (µU/mL) (*n* = 5391) Serum C-peptide (nmol/L) (*n* = 3668)	9.0 ± 0.2 0.75 ± 0.01	8.0 ± 0.2 0.70 ± 0.01	<0.0001 0.0001
HOMA-IR ^3^ (*n* = 5389) Serum C-reactive protein (µg/dL) (*n* = 8237) Serum total homocysteine (µmol/L) (*n* = 8227) Serum fibrinogen (mg/dL) (*n* = 1253) VO_2_ max (mL/kg/min) (*n* = 2625)	2.3 ± 0.04 149 ± 4 8.8 ± 0.1 347 ± 7 40 ± 0.2	1.9 ± 0.05 204 ± 6 7.5 ± 0.1 375 ± 5 28 ± 0.3	<0.0001 <0.0001 <0.0001 <0.0001 <0.0001

^1^ NHANES 2001–2002, NHANES 2003–2004, and NHANES 2005–2006 were concatenated into one analytic data file, NHANEs 2001–2006. Values are geometric mean ± SE. ^2^ Significance for the two-tailed unpaired t-test between men and women. Because serum 25(OH)D concentration, triglycerides, glucose, glycohemoglobin, insulin, C-peptide, C-reactive protein, Homeostatic Model Assessment-Insulin Resistance, and homocysteine were skewed, to satisfy the normality, natural logarithm transformed values were used. ^3^ Homeostatic Model Assessment-Insulin Resistance: Fasting insulin (µU/mL) × fasting glucose (mg/dL)/405.

**Table 2:** Multivariable adjusted odds ratio (OR) and 95% confidence interval (CI) for metabolic syndrome (MetS) according to clinical cut off serum 25-hydroxyvitamin D [25(OH)D] concentrations in US adults: National Health and Nutrition Examination Surveys (NHANES), 2001–2006 (*n* = 8241) ^1^.

Variable		Serum 25(OH)D Concentration			*p*-Value
	<30 nmol/L	30-<50 nmol/L	50-<75 nmol/L	≥75 nmol/L	
Sample size, *n*	1311	2570	3068	1292	
MetS Cases, *n*	621	1138	1144	323	
Prevalence of MetS (%)	45.5	41.7	34.5	21.6	<0.0001 ^2^
Unadjusted OR (95% CI)	3.03 (2.39, 3.84) ^3^	2.59 (2.05, 3.27) ^3^	1.91 (1.51, 2.43) ^3^	1.0 ^4^	<0.0001 ^5^
Multivariable adjusted OR (95% CI)	2.98 (2.14, 4.16) ^3^	2.84 (2.22, 3.64) ^3^	1.78 (1.38, 2.31) ^3^	1.0 ^4^	<0.0001 ^6^

^1^ Weighted *n* = 124,884,618. NHANES 2001–2002, NHANES 2003–2004, and NHANES 2005–2006 were concatenated into one analytic database, NHANESs 2001–2006. To convert serum 25(OH)D concentration nmol/L to ng/mL divide by 2.496. MetS was defined according to the modified National Cholesterol Education Program- Adult Treatment Panel III criteria, the presence of at least 3 of the following criteria: (1) waist circumference >102 cm for men and >88 cm for women, (2) triglycerides >150 mg/dL, (3) serum HDL cholesterol <40 mg/dL for men and <50 mg/dL for women, (4) systolic blood pressure >130 mm Hg or diastolic blood pressure >85 mm Hg or diagnosis of high blood pressure or use of medication for high blood pressure, and (5) fasting glucose >100 mg/dL or diabetes diagnosis or use of insulin or oral hypoglycemic medications. ^2^ Significance in the Rao-Scott χ^2^ test. ^3^ Significantly different from the referent category (*p* ≤ 0.05). ^4^ Referent category. ^5^ Significance for the effect of serum 25(OH)D concentration in the univariate logistic regression. ^6^ Significance for the effect of serum 25(OH)D concentration in the multivariable adjusted logistic regression. Data were adjusted for race-ethnicity, age, BMI, poverty income ratio, physical activity, and consumption of supplements. Interactions between serum 25(OH)D and BMI, serum 25(OH)D and race-ethnicity, age and BMI, race-ethnicity, and poverty income ratio were significant. Gender, season of blood draw, smoking status, and alcohol consumption were not significant in the model.

**Table 3:** Multivariable adjusted odds ratio (OR) and 95% confidence interval (CI) for diabetes risk according to clinical cut off serum 25-hydroxyvitamin D [25(OH)D] concentrations in US adults: National Health and Nutrition Examination Surveys (NHANESs), 2001–2006 (*n* = 12,194) ^1^.

Variable		Serum 25(OH)D Concentration			*p*-Value
	<30 nmol/L	30-<50 nmol/L	50-<75 nmol/L	≥75 nmol/L	
Sample size, *n*	1970	3816	4591	1817	
Diabetes Cases, *n* ^2^	283	465	372	100	
Prevalence, %	11.6	9.3	5.9	4.1	<0.0001 ^3^
Unadjusted OR (95% CI)	2.88 (2.14, 3.86) ^4^	2.28 (1.75, 2.98) ^4^	1.47 (1.16, 1.87) ^4^	1.0 ^5^	<0.0001 ^6^
Multivariable adjusted OR (95% CI)	1.70 (1.19, 2.43) ^4^	1.63 (1.20, 2.21) ^4^	1.33 (1.00, 1.78) ^4^	1.0 ^5^	0.008 ^7^

^1^ Weighted *n* = 182,793,002. NHANES 2001–2002, NHANES 2003–2004, and NHANES 2005–2006 were concatenated into one analytic data file, NHANES 2001–2006. To convert serum 25(OH)D concentration nmol/L to ng/mL divide by 2.496. ^2^ Status on diabetes was based on self-reported response to question on diabetes and who reported that they were on insulin therapy or taking oral hypoglycemic medications. This includes all cases of diabetes mellitus. ^3^ Significance in the Rao-Scott χ ^2^ test. ^4^ Significantly different from the referent category (*p* ≤ 0.05). ^5^ Referent category. ^6^ Significance for the effect of serum 25(OH)D concentration in the unadjusted cumulative logistic regression. ^7^ Significance for the effect of serum 25(OH)D concentration in the multivariable adjusted cumulative logistic regression. Data analysis was adjusted for age, race-ethnicity, BMI, poverty income ratio, and alcohol consumption. Interactions between serum 25(OH)D concentration and BMI, age and BMI, race-ethnicity and BMI were significant. Gender, season of blood draw, consumption of supplements, physical activity, and smoking were not significant in the model.

**Table 4:** Concentrations of cardiometabolic biomarkers according to the clinical cut off serum 25-hydroxyvitamin D [25(OH)D] concentrations in US adults: National Health and Nutrition Examination Surveys (NHANESs), 2001–2006 ^1^.

Variable		Serum 25(OH)D Concentration ^2^			*p* Value ^3^
	<30 nmol/L	30-<50 nmol/L	50->75 nmol/L	≥75 nmol/L	
Waist circumference, cm (*n* = 12047) ^4^	100 (98, 102) ^a^	100 (99, 101) ^a^	96 (95, 97) ^b^	93 (91, 94) ^c^	<0.0001
Systolic blood pressure, mm Hg (*n* = 11694) ^5^ Diastolic blood pressure, mm Hg (*n* = 11697) ^6^	124 (122, 125) 72.4 (71.1, 73.7) ^a^	123 (122, 123) 71.3 (70.6, 72.1) ^a^	122 (121, 123) 71.5 (70.8, 72.1) ^a^	121 (120, 122) 69.8 (68.9, 70.7) ^b^	0.12 <0.008
Serum HDL-cholesterol, mg/dL (*n* = 12172) ^7^ Serum LDL cholesterol, mg/dL (*n* = 5292) ^8^	53.7 (52.3, 55.2) ^a, b^ 116 (113, 119)	51.5 (50.7, 52.2) ^c^ 117 (114, 119)	53.2 (52.6, 53.9) ^a^ 118 (116, 119)	55.7 (54.8, 56.7) ^b^ 119 (116, 122)	<0.0001 0.45
Serum fasting triglycerides, mg/dL (*n* = 5451) ^9^	134 (117, 153) ^a^	130 (120, 140) ^a^	115 (110, 120) ^a,b^	107 (101, 112) ^b^	0.0001
Fasting plasma glucose, mg/dL (*n* = 5475) ^10^ Blood glycohemoglobin, % (*n* = 12179) ^11^ Serum insulin, µU/mL (*n* = 5434) ^12^ Serum C-peptide, nmol/L (*n* = 3707) ^13^ Serum total homocysteine, µmol/L (*n* = 12170)^14^	100 (98, 103) 5.49 (5.45, 5.54) ^a^ 11.4 (10.8, 12.1) ^a^ 0.78 (0.74, 0.82) ^a^ 8.78 (8.43, 9.15) ^a^	100 (99, 102) 5.48 (5.44, 5.51) ^a^ 10.9 (10.4, 11.5) ^a^ 0.76 (0.72, 0.80) ^a^ 8.22 (8.06, 8.38) ^b^	99 (98, 100) 5.44 (5.41, 5.47) ^a^ 9.5 (9.2, 9.9) ^b^ 0.71 (0.68, 0.74) ^b^ 7.87 (7.78, 7.96)^c^	97 (96, 99) 5.35 (5.31, 5.39) ^b^ 8.9 (8.6, 9.2) ^b^ 0.67 (0.66, 0.69) ^b^ 7.89 (7.74, 8.04) ^c^	<0.04 0.0002 <0.0001 <0.0001 <0.0001
Serum C-reactive protein, µg/dL (*n* = 11924) ^15^	222 (205, 241) ^a^	207 (197, 218) ^a,b^	191 (184, 198) ^b^	190 (179, 201) ^b^	<0.003
HOMA-IR (*n* = 5445) ^16^ Serum fibrinogen (mg/dL) (*n* = 1265) ^17^ VO_2_ max (mL/kg/min) (*n* = 2625) ^18^	2.50 (2.25, 2.78) ^a^ 368 (348, 388) 33.9 (33.2, 34.7) ^a^	2.22 (2.05, 2.41) ^a^ ^2^ 375 (362, 387) 33.6 (33.0, 34.2) ^a^	2.08 (1.98, 2.19) ^b^ 364 (355, 374) 34.4 (33.9, 34.9) ^a^	1.76 (1.67, 1.86) ^c^ 372 (358, 387) 35.8 (35.1, 36.6) ^b^	<0.0001 0.06 0.0009

^1^ NHANES 2001–2002, NHANES 2003–2004, and NHANES 2005–2006 were combined into one master database, NHANESs 2001–2006. To convert serum 25(OH)D concentration nmol/L to ng/mL divide by 2.496. ^2^ Multivariable adjusted serum 25(OH)D concentration means and their 95% confidence intervals. Multiple mean comparisons were made with Bonferroni correction test for those cardiometabolic variables using a family-wise significance level <0.05. Means with different superscript letters are significantly different from each other within the row if the contrast between two means has *p* < 0.0083 based on Bonferroni correction for six multiple comparisons (0.05/6). Lack of superscripts indicate non-significance of that cardiometabolic risk variable (in the row) in relation to serum 25(OH)D in the multivariable adjusted regression model. ^3^ Significance for the effect of serum 25(OH)D in the multivariable adjusted regression. ^4^ Data were adjusted for age, gender, race-ethnicity, physical activity, consumption of supplements, smoking status, and alcohol consumption, as well as for interactions between serum 25(OH)D and gender; serum 25(OH)D and race-ethnicity; race-ethnicity and gender; gender and alcohol consumption; and physical activity and alcohol consumption. ^5^ Data were adjusted for age, gender, race-ethnicity, season of blood draw, BMI, physical activity, and alcohol consumption, as well as for interactions between age and BMI; age and alcohol consumption; BMI and season of blood draw; and BMI and alcohol consumption. ^6^ Data were adjusted for age, gender, race-ethnicity, BMI, and smoking status, as well as for interactions between serum 25(OH)D and gender; and age and BMI. ^7^ Data were adjusted for age, gender, race-ethnicity, BMI, poverty income ratio, physical activity, consumption of supplements, smoking status, and alcohol consumption, as well as for interactions between serum 25(OH)D and gender; serum 25(OH)D and BMI; age and race-ethnicity; age and BMI; and smoking status and alcohol consumption. ^8^ Data were adjusted for age, gender, season of blood draw, and alcohol consumption, as well as for interaction between age and gender. ^9^ Data were adjusted for age, gender, race-ethnicity, BMI, physical activity, and smoking, as well as for interactions between serum 25(OH)D and age; serum 25(OH)D and race-ethnicity; serum 25(OH)D and BMI; age and gender; and age and BMI. Data analysis was performed on natural logarithmic transformed concentrations. ^10^ Data were adjusted for age, gender, race-ethnicity, BMI, season of blood draw, and consumption of supplements, as well as for interactions between serum 25(OH)D and age; and age and BMI. Data analysis was performed on natural logarithmic transformed values. ^11^ Data were adjusted for age, gender, race-ethnicity, BMI, season of blood draw, physical activity, consumption of supplements, and alcohol consumption, as well as for interactions between serum 25(OH)D and season of blood draw; age and race-ethnicity; age and alcohol consumption; gender and physical activity; and race-ethnicity and season of blood draw. Data analysis was performed on natural logarithmic transformed values. ^12^ Data were adjusted for age, gender, race-ethnicity, BMI, physical activity, consumption of supplements, smoking status, and alcohol consumption, as well as for interactions between age and gender; age and BMI; gender and BMI; BMI and consumption of supplements; and race and alcohol consumption. Data analysis was performed on natural logarithmic transformed values. ^13^ Data were adjusted for age, gender, race-ethnicity, BMI, season of blood draw, physical activity, and alcohol consumption, as well as for interactions between age and BMI; gender and race-ethnicity; and BMI and alcohol consumption. Data analysis was performed on natural logarithmic transformed values. ^14^ Data were adjusted for age, gender, race-ethnicity, poverty income ratio, season of blood draw, physical activity, consumption of supplements, smoking status, and alcohol consumption, as well as for interactions between serum 25(OH)D concentration and race; serum 25(OH)D and alcohol consumption, age and gender; age and poverty income ratio, age and smoking status; gender and race-ethnicity; and gender and smoking status. Data analysis was performed on natural logarithmic transformed values. ^15^ Data were adjusted for age, gender, race-ethnicity, BMI, physical activity, and smoking status, as well as for interactions between age and BMI; gender and BMI; gender and smoking status; and BMI and physical activity. Data analysis was performed on natural logarithmic transformed values. ^16^ Homeostatic Model Assessment-Insulin Resistance: Fasting insulin (µU/mL) × fasting glucose (mg/dL)/405. Data were adjusted for age, gender, race-ethnicity, BMI, physical activity, consumption of supplements, smoking status, and alcohol consumption, as well as for interactions between serum 25(OH)D concentration and age; age and BMI; gender and race-ethnicity; and race-ethnicity and BMI. Data analysis was performed on natural logarithmic transformed values. ^17^ Data were adjusted for age, gender, race-ethnicity, BMI, smoking status, and alcohol consumption, as well as for interaction between gender and alcohol consumption, and race-ethnicity and alcohol consumption. ^18^ Data were adjusted for age, gender, physical activity, and alcohol consumption, as well as for interaction between serum 25(OH)D concentration and gender.

## Abbreviations

1,25(OH)_2_D, 1,25-dihydroxyvitamin D; 25(OH)D, 25-hydroxyvitamin D; CI, confidence intervals; CRF, cardiorespiratory fitness; CVD, cardiovascular diseases; ES, Endocrine Society; H/MA, Hispanic/Mexican American; HOMA-IR, homeostatic model assessment-insulin resistance; IOM, Institute of Medicine; MEC, mobile examination center; MetS, metabolic syndrome; NCHS, National Center for Health Statistics; NHANES, National Health and Nutrition Examination Survey; NHB, non-Hispanic black, NHW, non-Hispanic white; OR, odds ratio; PIR, poverty income ratio; RCTs, randomized controlled trials; STROBE, Strengthening of Reporting of Observational Studies in Epidemiology; VDR, vitamin D receptors; VO_2_ max, maximal oxygen uptake.
